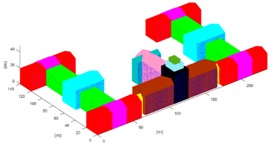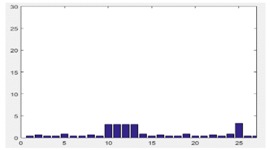# A Dynamic Multi-Projection-Contour Approximating Framework for the 3D Reconstruction of Buildings by Super-Generalized Optical Stereo-Pairs

**DOI:** 10.3390/s17092153

**Published:** 2017-09-19

**Authors:** Yiming Yan, Nan Su, Chunhui Zhao, Liguo Wang

**Affiliations:** Department of Information and Communication Engineering, Harbin Engineering University, Harbin 150001, China; zhaochunhui@hrbeu.edu.cn (C.Z.); wangliguo@hrbeu.edu.cn (L.W.)

**Keywords:** remote sensing, 3D reconstruction, super-generalized stereo-pairs, artificial bee colony algorithm

## Abstract

In this paper, a novel framework of the 3D reconstruction of buildings is proposed, focusing on remote sensing super-generalized stereo-pairs (SGSPs). As we all know, 3D reconstruction cannot be well performed using nonstandard stereo pairs, since reliable stereo matching could not be achieved when the image-pairs are collected at a great difference of views, and we always failed to obtain dense 3D points for regions of buildings, and cannot do further 3D shape reconstruction. We defined SGSPs as two or more optical images collected in less constrained views but covering the same buildings. It is even more difficult to reconstruct the 3D shape of a building by SGSPs using traditional frameworks. As a result, a dynamic multi-projection-contour approximating (DMPCA) framework was introduced for SGSP-based 3D reconstruction. The key idea is that we do an optimization to find a group of parameters of a simulated 3D model and use a binary feature-image that minimizes the total differences between projection-contours of the building in the SGSPs and that in the simulated 3D model. Then, the simulated 3D model, defined by the group of parameters, could approximate the actual 3D shape of the building. Certain parameterized 3D basic-unit-models of typical buildings were designed, and a simulated projection system was established to obtain a simulated projection-contour in different views. Moreover, the artificial bee colony algorithm was employed to solve the optimization. With SGSPs collected by the satellite and our unmanned aerial vehicle, the DMPCA framework was verified by a group of experiments, which demonstrated the reliability and advantages of this work.

## 1. Introduction

In recent years, there has been a dramatic proliferation of research concerned with three dimensional (3D) reconstruction of buildings by remote sensing resources. Data collected by different sensors could be used finishing this work, like optical images [1], synthetic aperture radar (SAR) [2], and light detection and ranging (LiDAR) [3]. There are also some multi-data source and multi-sensor approaches [4,5]. Although higher precision 3D points of a city area can be obtained by LiDAR, it is an inadequate resource for certain applications, such as rescue and anti-terrorism reconnaissance. Undisputedly, optical images are the most adequate remote sensing resources. Super-generalized stereo-pairs (SGSPs) can be defined as two or more optical images, which have overlapping coverage areas and might have different resolutions, collected by the same or different sensors at diverse times and views. As we all know, a classic 3D reconstruction framework is based on stereo-pairs collected by spaceborne or airborne [6,7]. These stereo-pairs are required to satisfy certain restrictions (convergence angle, base-to-height ratio, etc.) to perform better stereo matching and calculation of 3D coordinates [8]. However, SGSPs might not afford these restrictions, and stereo matching is undoubtedly the technical bottleneck when doing 3D reconstruction by SGSPs [9,10]. People always failed to generate digital elevation/surface model of regions of buildings, and cannot do further 3D shape modeling for the buildings. Although some gradually developing products, like smart 3D and Street Factory, generate dense 3D point clouds using state-of-the-art pixel-based multi-stereo image matching [11], an amount of manual correction is needed to enhance the geometric detail of 3D building models. In other words, the 3D reconstruction of buildings, using SGSPs, cannot be well achieved by traditional frameworks.

Here, we introduce a dynamic multi-projection-contour approximating (DMPCA) framework to reconstruct the 3D shape of buildings using SGSPs.

## 2. The Dynamic Multi-Projection-Contour Approximating Framework

**Figure 1:**
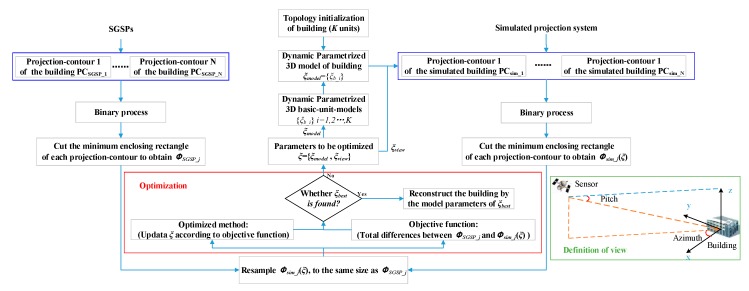
The 3D reconstruction of a building based on a framework of dynamic multi-projection-contour approximating.

The main framework of DMPCA is shown in Figure 1. The key idea is that in each of the specific views of SGSPs, we approximate the projection-contour of the building in the optical images by the projection-contour of a simulated 3D model of that building. Specifically, we establish an optimization to find a group of parameters of a simulated 3D model. It minimizes the total differences between projection-contour of a building in SGSPs and projection-contours of the simulated 3D model. Then, the simulated 3D model, defined by the optimized parameters, dynamically approximate the actual 3D shape of the building.

The work here is based on the hypothesis as expressed in Equation (1). Assume that a simulated 3D shape *S_sim_* is close to the actual 3D shape of building *S_true_*, when two or more projection-contours (PC) *PC_SGSPs_* from actual images are approximated by the corresponding *PC_sim_* (*N* is the number of images of SGSPs; *PC_SGSP_* is the projection-contour of the building after imaging; *PC_sim_* is the projection-contour of the building generated by *S_sim_*).

(1) $$S_{sim}\overset{}{\to }S_{true},when\;\underset{j=1,2,\ldots ,N}{PC_{sim\_j}\to PC_{SGSP\_j}}$$

(2) $$\begin{array}{ll} \min & f(\Phi_{SGSP\_j},\Phi_{sim\_j}(\xi ))j=1,2,\ldots ,N\\ & \xi =\text{\{}\xi_{model},\xi_{view}{}_{\_j}\text{\}} \end{array}$$

We performed the optimization as expressed in Equation (2). *Φ_SGSP_j_* is a feature image after binary and cutting processing of *PC_SGSP_j_*. The cutting area is the minimum enclosing rectangle (MER) of the projection-contour of the building. Meanwhile, *Φ_sim___j_*(*ξ*) is the simulated feature image after binary and cutting processing of *PC_sim_j_*(*ξ*), which need to be generated from the dynamic simulated 3D model, cut by the MER of the projection-contour of building, and resampled to the same size as the corresponding *Φ_SGSP_j_*.

Elements of *ξ* are divided into two parts: (1) parameters of the simulated 3D basic-unit-models *ξ_model_*, such as length, width, and height, which define the structure of buildings; and (2) parameters of the view *ξ_view_j_ =* {Azimuth*__j_*, Pitch*__j_*}, which are the imaging angle of the *j*-th image of SGSPs, defined as in Figure 1. If *ξ_view_j_* values are known, it can be preset. Moreover, if one or more Azimuth*__j_* and Pitch*__j_* are roughly known, we need to initial values of *ξ_view_j_*, and optimize them to find the matching view of the corresponding SGSP for more precise generation of *Φ_sim___j_*(*ξ*).

The difference between each *Φ_SGSP_j_* and the dynamic *Φ_sim___j_*(*ξ*) need to be compared whenever the parameter-vector *ξ* changed, and obtain the objective function *f*(*Φ_SGSP_j_*, *Φ_sim___j_*(*ξ*)). An optimized algorithm is employed to search *ξ_best_* by iteration, and then use *ξ_model_* of *ξ_best_* to reconstruct the 3D shape of the building.

### 2.1. Parameterized 3D Simulated Models and Projection

**Figure 2:**
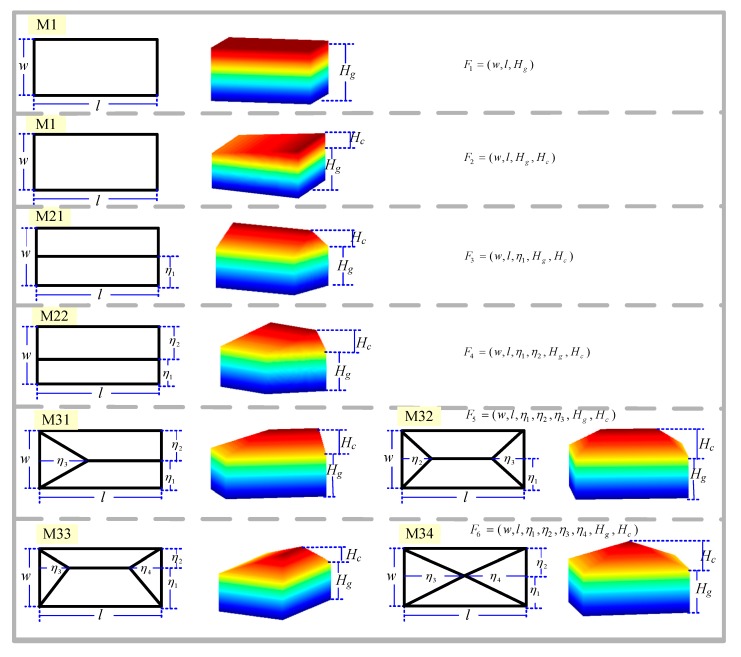
Typical basic-unit-models.

To obtain the dynamic *Φ_sim___j_*(*ξ*), groups of parameterized 3D models are needed. Inspired by [12], some typical parameterized 3D basic-unit-models were designed that could fit most cases of ridge buildings, as shown in Figure 2. Expressed by Equation (3), it is a universal model for these types of buildings. The basic-unit-model could be expanded to fit different applications if necessary.

(3) $$F(\xi_{model})=F(w,l,\eta_{1},\eta_{2},\eta_{3},\eta_{4},H_{g},H_{c}).$$

Commonly, a simple 3D shape can be described using these basic-unit-models. A complex 3D shape needs to be described by a group of basic-unit-models in a certain topological initial state. *ξ_model_* = {*ξ_b_i_*}, *i* = 1, 2, 3, …, *K*, *K* is a number of basic-unit-models.

With the parameterized 3D model, we set the entire model *ξ_model_* at the center of the simulated 3D coordinate system. Then, the projection-image of the 3D model of any view could be simulated by Equation (4), using some integrated functions of 3D visualization according to the simulated imaging model *V*. For aerial images, the perspective projection model is employed, and a generalized model, such as the rational function model (RFM), is used for satellite images. Whenever the parameter *ξ* is changed, the 3D model on the screen is shown by the 3D visualization functions, and the screenshots are saved as the simulated projection.

(4) $$PC_{sim\_j}\left(\xi \right)=V(M(\xi_{model}),\xi_{view\_j}).$$

### 2.2. Dynamic Multi-Projection-Contour Approximating by an Artificial Bee Colony Algorithm

In both *PC_SGSP_* and *PC_sim_*, the building areas are set to 1, while other parts are set to 0. We then cut the *MER* to obtain *Φ_SGSP_j_* and *Φ_sim___j_*(*ξ*). *Φ_sim___j_*(*ξ*) is then resampled to the same size as *Φ_SGSP_j_*, and Equation (5) is employed as an objective function. Similarity *S_Ö_* or normalized similarity *Ŝ_Ö_* can be used to define the similarity between all *Φ_SGSP_j_* and *Φ_sim___j_*(*ξ*) values, as expressed in Equation (6). Then, an artificial bee colony (ABC) algorithm can be employed to resolve the optimization.

The artificial bee colony algorithm groups the bee colony into three roles: employed bees (EB), onlooker bees (OB), and scout bees (SB), which are also the three phases of the algorithm. The main process is as follows:

(5) $$\begin{array}{l} f=-S_{\Phi }(\Phi_{SGSP\_j},\Phi_{sim\_j}(\xi ))\\ \xi =\underset{i=1,2,\ldots ,K}{\text{\{}\xi_{model},\xi_{view\_j}\text{\}}=\{w_{i},l_{i},\eta_{i}{}_{1},\eta_{i}{}_{2},\eta_{i}{}_{3},\eta_{i}{}_{4},H_{i}{}_{g},H_{i}{}_{c},Azimuth_{\_j},Pitch_{\_j}\}} \end{array}$$

(6) $$S_{\Phi }=\sqrt{\sum_{j=1}^{N}{\left(\frac{\Phi_{SGSP\_j}\text{\&}\Phi_{sim\_j}(\xi )}{\Phi_{SGSP\_j}|\Phi_{sim\_j}(\xi )}\right)}^{2}}\begin{array}{cc} or & {\hat{S}}_{\Phi }=\frac{1}{N}\sqrt{\sum_{j=1}^{N}{\left(\frac{\Phi_{SGSP\_j}\text{\&}\Phi_{sim\_j}(\xi )}{\Phi_{SGSP\_j}|\Phi_{sim\_j}(\xi )}\right)}^{2}} \end{array}.$$

(1) Parameter Initialization of the ABC Algorithm

Population number (*PN*), dimension of *ξ* (*D*), triggering threshold of scout bee (*TTSB*), maximum cycle number (*MCN*), or ideal fitness threshold (*IFT*) can be set for termination.

(2) Initialization of Solutions

The solution vector *ξ* is composed by all parameters to be optimized. Initiate *PN*/2 solutions *ξ_m_*(*n*) (*m* = 1, 2, …, *PN*/2, *n* = 1, 2, …, *D*) randomly according to predefined logical ranges for each element of *ξ*. *PN*/2 failure counters *FC_m_* are reset for each solution.

(3) Iteration and Modify all *PN*/2 Solutions

1) The EB phase. Each EB corresponds to a solution. Each solution *ξ_m_*(*n*) is modified by Equation (7), where *ξ_k_*(*n*) is another solution in the *PN*/2 solutions, *λ_m_*(*n*)~U[−1, 1].
  (7) $${\bar{\xi }}_{m}^{}(n)=\xi_{m}^{}(n)+\lambda_{m}^{}(n)\cdot (\xi_{m}^{}(n)-\xi_{k}^{}(n)).$$
  To improve the efficiency of the optimization, instead of modifying one element randomly in the original algorithm, all *D* elements of *ξ_m_* are modified. *f*(*ξ_m_*) and *f*(${\bar{\xi }}_{m}^{}$) are compared, and the better one is reserved to update the solution of this EB. The *FC_m_* will add one if *f*(*ξ_m_*) > *f*(${\bar{\xi }}_{m}^{}$); otherwise, it remains unchanged.
2) The recruiting probability of each EB is estimated using Equation (8).
  (8) $$prob(m)=f(\xi_{m})/\sum_{m=1}^{PN/2}f(\xi_{m}).$$
3) The OB phase: OB optimizes the solution found by the EB it follows. Each OB follows one EB according to recruiting probability, and the solution of that EB is modified. Since better solutions having greater recruiting probability, they can be optimized more times. The modification is the same as the EB phase.
4) The best solution *ξ_best_* is recorded. All the updated *PN*/2 solutions are ranked according to their fitness, and *ξ_best_* is recorded. Then, if the algorithm comes to the *MCN* or *f*(*ξ_best_*) reaches *IFT*, it ends. *ξ_model_* of the *ξ_best_* is chosen for further reconstruction.
5) The SB phase: SB is triggered only if *FC_m_* reaches *TTSB*, the corresponding solution is abandoned, and a new solution will be randomly generated and the original one replaced.

## 3. Experiments

In the experiments, the same ABC parameters are used to fairly test the performance of the DMPCA in different SGSP cases. The programs were run in MATLAB 2015, and the computer is with Interl(R) Core(TM) i5-6200 2.3GHz and 8G RAM. Considering the computing ability and time-consuming problem, the main parameters of the ABC algorithm were set as *PN* = 10, *TTSB =* 50, and *MCN* = 100.

### 3.1. An Analysis of 3D Reconstruction by DMPCA Using Simulated SGSPs

Firstly, we intended to find the relationship between similarity *S_Ö_* and the precision of the reconstruction.

**Figure 3:**
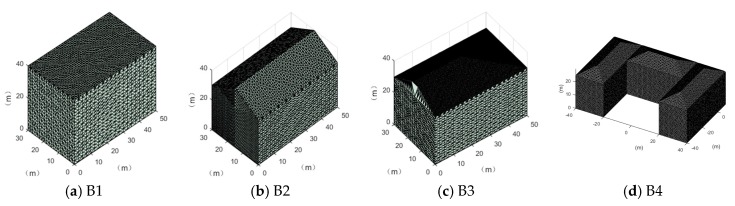
Typical simulated 3D buildings. (**a**–**c**) are three single buildings with different roofs; (**d**) is a combined one.

Since imaging angles of the actual remote sensor cannot be precisely controlled, we picked three typical buildings with different 3D shapes, as shown in Figure 3 and Table 1, and simulated their SGSPs with accurate imaging angles, as shown in Table 2. The resolution of the SGSPs is 1 m/pixel. For convenience, azimuth and pitch angles are defined according to the center of the buildings, as shown in the bottom-right section of Figure 1.

Due to the symmetry of the three buildings, we picked azimuth and pitch angles of 20 regular simulated images (RSI) in 0~90°, and two special simulated images (SSI) are (150°, 45°) and (225°, 45°). Then, we chose one SSI together with any RSI to constitute SGSPs for 3D reconstruction. We compared the error of reconstruction by Equation (9), where *NT* is the number of testing points, and we compared the actual coordinate (*X_n_*, *Y_n_*, *Z_n_*) with the estimated ones (${\hat{X}}_{n}$, ${\hat{Y}}_{n}$, ${\hat{Z}}_{n}$).

(9) $$PRE=\frac{1}{NT}\sum_{n=1}^{NT}\sqrt{{\left({\hat{X}}_{n}-X_{n}\right)}^{2}+{\left({\hat{Y}}_{n}-Y_{n}\right)}^{2}+{\left({\hat{Z}}_{n}-Z_{n}\right)}^{2}}$$

**Figure 4:**
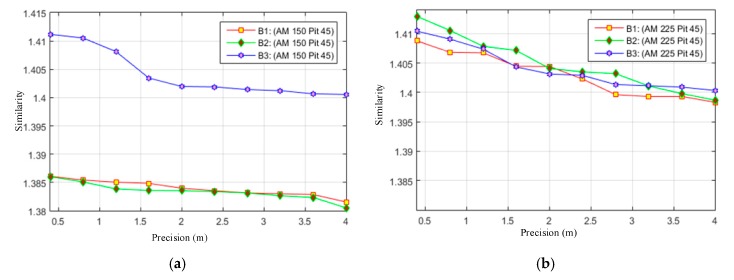
“Precision” versus “***S_Φ_***”. “Precision” is obtained by Equation (9) with all sampled points of the roof of the buildings. “Similarity” is the average minimum ***S_Φ_*** when reconstructed by different SGSPs. (**a**) has the specific view (150°, 45°), while (**b)** has the specific view (225°, 45°).

We first tested the minimum requirements of similarity *S_Ö_* for different precisions of 3D reconstruction by our DMPCA method. It can be seen in Figure 4a,b that similarity *S_Ö_*, defined by the feature-image *Φ*, can be an effective restriction of the 3D shape, and higher precision requires a larger *S_Ö_* from the trend for different SGSP combinations. Nevertheless, in the same precision, the requirements of *S_Ö_* are different for different buildings, also for different cases of SGSPs.

**Figure 5:**
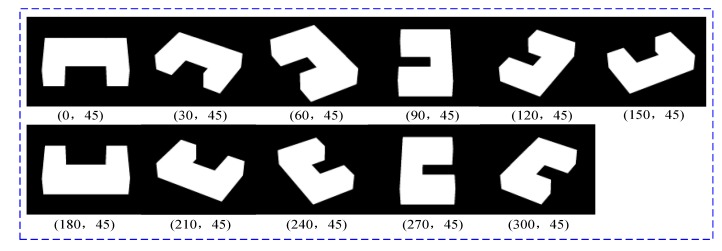
The simulated projections of B4 in different views.

Two views were used in the experiments of B1~B3, and the three buildings are entirely symmetrical. Furthermore, B4 was used, which is not perfectly symmetrical but was a complex building. Then, the performance of DMPCA on B4 was tested in different combination cases of three different views;

We focused on the effects of the azimuth spans of DMPCA, and the pitch angles of input images were fixed at 45°. Eleven views of B4 were used, as shown in Figure 5. First, the cases of equidistant azimuth spans were considered. The initial azimuth was set to 0°, and the azimuth spans between two adjoined images were set to five cases (0°, 30°, 45°, 60°, 90°, and 120°), so the combinations of azimuths of the five cases were as follows: 0-30-60, 0-45-90, 0-60-120, 0-90-180, and 0-120-240. The results after 100 iterations are shown in Table 3. It could be found that, in the case of equidistant azimuth spans, a larger azimuth span produces better precision.

**Figure 6:**
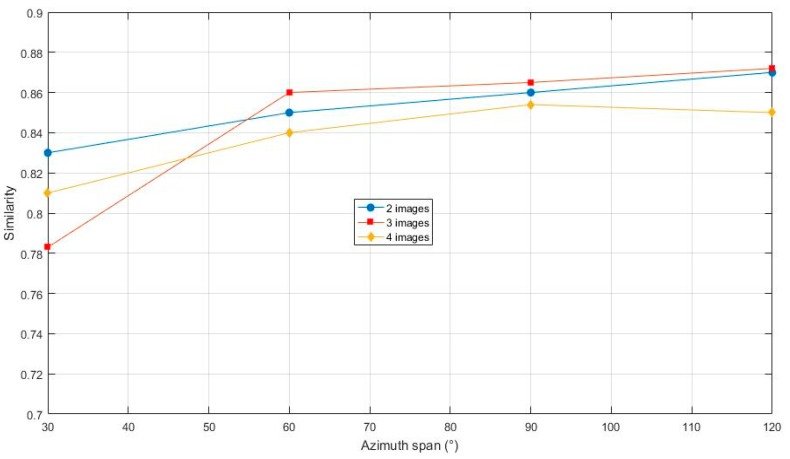
Results of different number of views.

Moreover, also in equidistant azimuth spans, two-view cases and four-view cases were tested, similar to the above three-view cases (note that, when using four views of the 120° azimuth span, two of the four were entirely overlapped; this input case is the same as the case using three views of the 120°, but the one redundancy image deduced the optimizing process and, compared with the case using three views, obtained a worse result). It was illustrated that three views are the better choice in equidistant azimuth spans, as shown in Figure 6.

**Figure 7:**
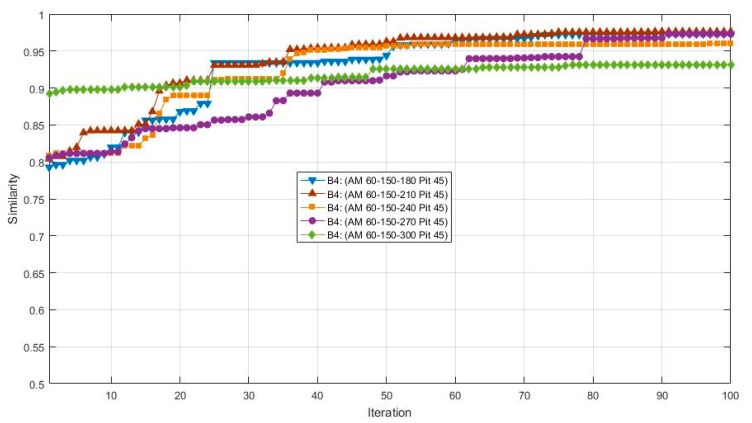
The results of cases (60-150-X, 45) in 100 iterations.

**Figure 8:**
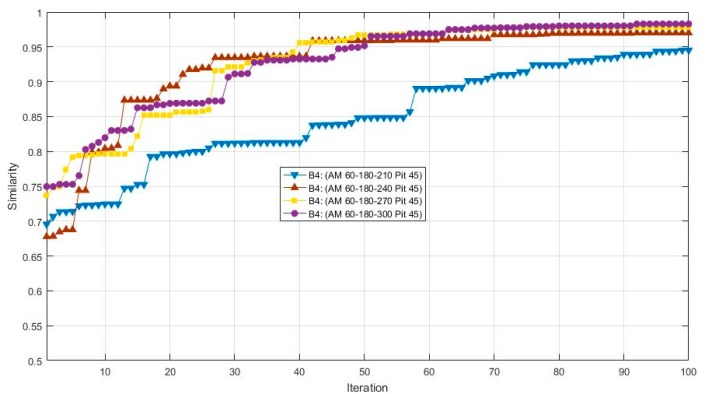
The results of cases (60-180-X, 45) in 100 iterations.

According to a number of experiments, it was found the initial azimuth also has certain effects on the results, since the results of a 0° initial azimuth is no better than other cases of initial azimuth. The initial azimuth was then set to 60°, and the cases of non-equidistant azimuth spans were tested. The views and results are shown in Table 4, Figure 7 and Figure 8. After 100 iterations, the 60-150-300 case obtained the highest value of similarity and precision. Moreover, it can be found that similarity and precision do not increase simultaneously.

It can be concluded from above that the complementarity of the azimuth is the most significant factor of the DMPCA method.

### 3.2. 3D Reconstruction by DMPCA Using Actual Remote Sensing SGSPs

**Figure 9:**
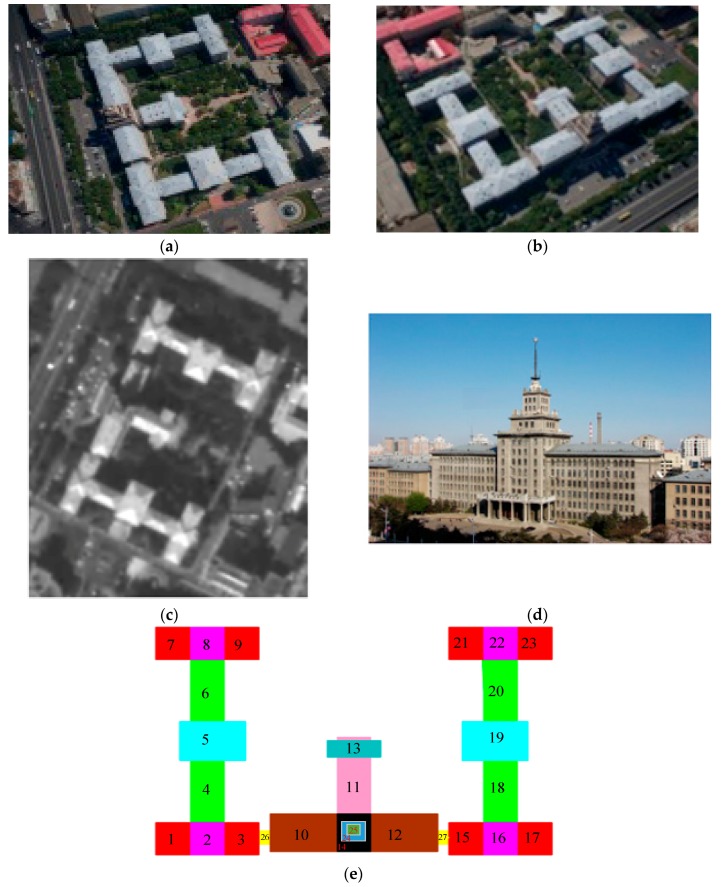
SGSPs of the main building of HIT. (**a**,**b**) are two airborne images, the resolution is about 0.4 m/pixel; (**c**) is a satellite image, the resolution is 2 m/pixel; (**d**) is the official display of the building. (**e**) is the schematic diagram of the initialized 27 topological units.

We tried to reconstruct the main building of the Harbin institute of technology (HIT). As shown in Figure 9, we collected a satellite image (SI) from a vendor and two aerial images (AI1, AI2) by our unmanned aerial vehicle.

It could be found that aerial images are oblique, while satellite images are nadir-looking. Moreover, compared to AI2, SI and AI1 have similar azimuths. We combined them two by two and obtained three groups of SGSPs (SGSP1 = SI & AI1, SGSP2 = SI & AI2, SGSP3 = AI1 & AI2). Then, we reconstructed the building using the three groups of SGSPs, respectively.

The projection-contours of the building were extracted from each SGSPs using the image matting methods [13] and some manual correction. Since the building has a complex 3D shape, we initialized it into 27 units (*ξ_model_* = {*ξ_b_i_*}, *i* = 1, 2, …, 27) as Figure 9e, including 25 outer-units and 2 inner-units. For each basic unit, the horizontal parameters were resolved from the SI referring to the building extraction methods in [14]. Then, the height parameters of 27 basic-unit-models and view parameters were collected to compose *ξ*. Note that we were approximating the projection-contour of the entire model of the building, not each single unit. Therefore, the objective function *f* in Equation (5) needs no changes.

According to the design diagram of this building, we collected the true-value of the top of the building. Each reconstructed unit was sampled into 0.1 *m* interval 3D points-cloud, and we selected all the sampled points of the roof of building as testing points, to test the precision by Equation (9). The results are shown in Table 5.

The results revealed that, overall, 3D reconstruction of the building could be achieved by any of the three SGSPs using our DMPCA. A larger *S_Ö_* produces better results, which support our hypothesis as shown in Equation (1). In fact, a larger *S_Ö_* is sufficient but not necessary for better reconstruction, since our simulated perspective projection model cannot fit the actual imaging model perfectly. The aerial SGSP3 with higher resolution produces better precision than SGSP1 and SGSP2. Particularly, although the precision of several units obtained by SGSP2 are poorer than that obtained by SGSP1, these units are in small proportion of the entire building, so SGSP2 obtained better mean precision than SGSP1. The only difference between SGSP1 and SGSP2 is the azimuth of AI1 and AI2. We think that this made SGSP2 a better complementarity with SI. This indicated that the precision of 3D reconstruction by our DMPCA framework mainly depends on the resolution and complementarity of the imaging angle of the SGSPs. Moreover, the precision of each unit, obtained by the respective SGSPs, is different. The inner-units could not be effectively restricted by the projection-contour, since there is only a little piece of part or none of the inner-units in the projection-contour. Thus, the precision of the inner-units cannot be guaranteed, which mostly depends on the pitch angle of the view. However, most of the outer-units have an ideal precision according to their inputting SGSPs.

## 4. Discussion and Conclusions

A dynamic multi-projection-contour approximating (DMPCA) framework was proposed in this paper. Using super-generalized stereo-pairs (SGSPs), the reconstruction of the 3D shape of a building was achieved, while traditional frameworks failed. In this framework, we resolved an optimization to find the best parameters of a certain group with a simulated 3D model, which minimizes differences between the projection-contour of a building in the actual optical images and that of the simulated 3D model. According to experiments, we found that the proposed similarity can well restrict the shape of the building. However, the similarity and precision do not increase simultaneously. There are two possible reasons: (1) our projection model is not absolutely consistent with the actual imaging model; (2) the optimizing algorithm did not find the best solution of the parameters. Moreover, the effect of azimuth spans on DMPCA were analyzed. In the equidistant azimuth spans cases, we found that a greater number of views will not produce a better result, and three views could be a suitable choice. In the non-equidistant azimuth spans cases, it was proved that the azimuth spans, which has better complementarity, can improve the performance of DMPCA, since they have better restriction of the shape. In the test of actual SGSPs, the precision of 3D reconstruction by our DMPCA mainly depends on the resolution and complementarity of the imaging angle of the SGSPs. A higher resolution and better complementarity of azimuth produce better precision.

Furthermore, to improve this work, a basic-unit-model could be better defined for greater application, and the optimized algorithm could be improved to enhance the performance.

## Figures and Tables

**Table 1:** Typical buildings (unit: m).

Buildings	*l*	*W*	*η*_1_	*η*_2_	*η*_3_	*η*_4_	*H_G_*	*H_C_*
B1	50	30	0	0	0	0	30	0
B2	50	30	15	15	0	0	30	10
B3	50	30	15	15	25	0	30	10
B4_L	50	20	10	10	10	10	20	5
B4_R	50	20	10	10	10	10	20	5
B4_M	40	20	10	10	10	10	20	5

**Table 2:** Views of simulated images (unit: °).

Form of View: (Azimuth*_SGSP_*, Pitch*_SGSP_*)				
(0, 30)	(0, 45)	(0, 60)	(0, 75)	(0, 90)
(30, 30)	(30, 45)	(30, 60)	(30, 75)	(30, 90)
(60, 30)	(60, 45)	(60, 60)	(60, 75)	(60, 90)
(90, 30)	(90, 45)	(90, 60)	(90, 75)	(90, 90)
Special:	(150, 45)		(225, 45)	

**Table 3:** Performance of DMPCA on different cases of equidistant azimuth spans.

Azimuth Spans	Similarity	Precision (m)
30°	0.815	1.73
45°	0.822	1.66
60°	0.841	1.56
90°	0.872	1.37
120°	0.923	1.07

**Table 4:** The performance of DMPCA on different cases of non-equidistant azimuth spans.

The Azimuths	Similarity	Precision (m)
60-150-180	0.930	0.8400
60-150-210	0.961	0.4800
60-150-240	0.975	0.3000
60-150-270	0.976	0.2880
60-150-300	0.988	0.1440
60-180-210	0.948	0.526
60-180-240	0.969	0.512
60-180-270	0.973	0.5115
60-180-300	0.985	0.5085

**Table 5:** Reconstruction results obtained by different SGSPs. “Precision” is the mean PRE of the 27 basic-unit-models. Figures in the last column show the error of 3D reconstruction; the *x*-axis is the serial number of units, and the *y*-axis is PRE (unit: m).

SGSPs	Precision	Visualization of the 3D Reconstruction	Error of 3D Reconstruction
SGSP1	3.1 (m)	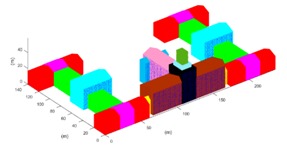	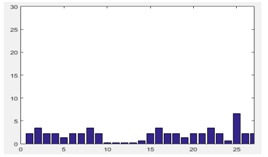
SGSP2	2.1 (m)	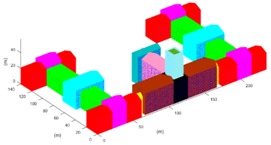	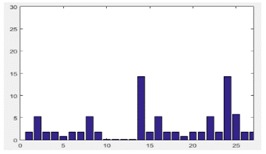
SGSP3	1.0 (m)