# Correction: Yan, Y.; et al. A Dynamic Multi-Projection-Contour Approximating Framework for the 3D Reconstruction of Buildings by Super-Generalized Optical Stereo-Pairs. *Sensors* 2017, *17*, 2153

**DOI:** 10.3390/s19010191

**Published:** 2019-01-07

**Authors:** Yiming Yan, Nan Su, Chunhui Zhao, Liguo Wang

**Affiliations:** College of Information and Communication Engineering, Harbin Engineering University, Harbin 150001, China; zhaochunhui@hrbeu.edu.cn (C.Z.); wangliguo@hrbeu.edu.cn (L.W.)

The authors wish to make the following corrections to this paper [1]:

Change in Acknowledgement

Due to a lapse, the Acknowledgement section was missing from the original article version [1].

**Acknowledgments:** The authors would like to thank the support from the Fund of the National Natural Science Foundation of China under Grant No. 61601135 and Natural Science Foundation of Heilongjiang Province of China under Grant No. QC201706802.

The authors would like to apologize for any inconvenience caused to the readers by these changes.
